# Treatment outcome and survival for HIV and AIDS patients coinfected with Mycobacterium Tuberculosis in Nava Kiran Super Speciality Center for AIDS care in Nepal

**DOI:** 10.1186/1742-4690-9-S1-P140

**Published:** 2012-05-25

**Authors:** Ujjwal Baral, Prakash Yogi, Anita Pradhan, Ramesh Pandey

**Affiliations:** 1 National Association of People Living With Hiv and Aids in Nepal, Kathmandu, Nepal

## Topic

Treatment outcome and survival for HIV and AIDS patients coinfected with Mycobacterium Tuberculosis in Nava Kiran Super Speciality Center for AIDS care in Nepal.

## Introduction

Tuberculosis (TB) is a frequent disease among People Living with HIV/ AIDS (PLHAs) in Nepal.Prevalence is more than 70% and TB is still a major killer in PLHAs and first Oppertunistic Infection (OI) among PLHAs. We intend to assess prevalence, clinical features, treatment outcome, resistant to first line antitubercular drugs and survival of PLHAs with TB.

## Method

A longitudinal retrospective-prospective study of clinical records from co-infected before and after Antiretroviral Treatment (ART) with confirmed TB diagnosis or clinical suspect was done. Sensitivity of drugs performed in cases of Treatment failure or epidemiological issues. Treatment success was defined as cure; poor outcome included incomplete treatment, failure or death.

## Results

8913 patients since 2007 to 2009 admitted at Nava Kiran Super Speciality Center, 680 clients were co-infected. TB incidence was 68.9%. Age ranged from 16 to 61 years, 75% were under 40 years and 520 clients that is 76.47% were male. HIV infection route; intravenous drug users 59.26%, heterosexual 28.32%, MSM 8.15%, and others were 28.32%. Mean CD4 was 150 cells/cml. TB was first incident in 59.6%. Pulmonary TB account 590 (86.76%) with acid fast smear positive in 87% and extrapulmonary TB counted 90 (13.23%). Multiple Drug Resistant (MDR) TB account 52 (8.8%) of pulmonary TB. In pulmonary TB complete cure was obtained in 64.03% and 22.97% in MDR TB inspite of adequate treatment. Success was high in Pulmonary TB.

## Conclusion

TB is devastating situation in PLHAs of Nepal. A high suspect, early diagnosis and documented sensitivity improve the outcome and diminished sequel and mortality.

